# Prognostic Factors for Cervical Spinal Cord Injury without Major Bone Injury in Elderly Patients

**DOI:** 10.1089/neu.2021.0351

**Published:** 2022-04-20

**Authors:** Hideaki Nakajima, Noriaki Yokogawa, Takeshi Sasagawa, Kei Ando, Naoki Segi, Kota Watanabe, Satoshi Nori, Shuji Watanabe, Kazuya Honjoh, Toru Funayama, Fumihiko Eto, Yoshinori Terashima, Ryosuke Hirota, Takeo Furuya, Tomohiro Yamada, Gen Inoue, Takashi Kaito, Satoshi Kato

**Affiliations:** ^1^ Department of Orthopaedics and Rehabilitation Medicine, Faculty of Medical Sciences University of Fukui, Fukui, Japan.; ^2^ Department of Orthopaedic Surgery, Graduate School of Medical Sciences, Kanazawa University, Ishikawa, Japan.; ^3^ Department of Orthopedics Surgery, Toyama Prefectural Central Hospital, Toyama, Japan.; ^4^ Department of Orthopedic Surgery, Nagoya University Graduate School of Medicine, Nagoya, Japan.; ^5^ Department of Orthopaedic Surgery, Keio University School of Medicine, Tokyo, Japan.; ^6^ Department of Orthopaedic Surgery, Faculty of Medicine, University of Tsukuba, Ibaraki,Japan.; ^7^ Department of Orthopaedic Surgery, Graduate School of Comprehensive Human Sciences, University of Tsukuba, Ibaraki, Japan.; ^8^ Department of Orthopaedic Surgery, Sapporo Medical University, Sapporo, Japan.; ^9^ Department of Orthopaedic Surgery, Matsuda Orthopedic Memorial Hospital, Sapporo, Japan.; ^10^ Department of Orthopaedic Surgery, Graduate school of Medicine, Chiba University, Chiba, Japan.; ^11^ Department of Orthopaedic Surgery, Hamamatsu University School of Medicine, Shizuoka, Japan.; ^12^ Department of Orthopaedic Surgery, Nagoya Kyoritsu Hospital, Aichi, Japan.; ^13^ Department of Orthopaedic Surgery, Kitasato University School of Medicine, Kanagawa,Japan.; ^14^ Department of Orthopaedic Surgery, Osaka University Graduate School of Medicine,Osaka, Japan.

**Keywords:** cervical spinal cord injury, elderly patients, multi-variate analysis, neurological improvement, no major bone injury, prognostic factor

## Abstract

In the current aging society, there has been a marked increase in the incidence of cervical spinal cord injury (CSCI) without major bone injury. This multi-center study aimed to identify predictors of neurological improvement in elderly patients with CSCI without major bone injury. The participants were 591 patients aged ≥65 years with CSCI without major bone injury and a minimum follow-up period of three months. Neurologic status was defined using the American Spinal Injury Association (ASIA) impairment scale (AIS). Univariate and multi-variate analyses were performed to identify prognostic factors for walking recovery in AIS A–C cases and full upper extremity motor recovery in AIS D cases. In AIS A–C cases, body mass index (odds ratio (OR): 1.112), magnetic resonance imaging signal change (OR: 0.240), AIS on admission (OR: 3.497), comorbidity of dementia/delirium (OR: 0.365), and post-injury pneumonia (OR: 0.194) were identified as independent prognostic factors for walking recovery. The prevalence of ossification of the posterior longitudinal ligament (OR: 0.494) was also found to be an independent prognostic factor in AIS B and C cases only. In AIS D cases, age (OR: 0.937), upper extremity ASIA motor score on admission (OR: 1.230 [per 5 scores]), and operation (OR: 0.519) were independent prognostic factors for full motor recovery. The severity of AIS at admission was the strongest predictor of functional outcomes. Promoting rehabilitation, however, through measures to reduce cognitive changes, post-injury pneumonia, and unhealthy body weight changes can contribute to greater neurological improvement in AIS A–C cases.

## Introduction

Spinal cord injury (SCI) may result in loss of neurological function, which can be life-changing and economically damaging for a patient. A systematic review found an incidence of SCI of 3.6 to 195.4 per million worldwide,^1^ and a comprehensive nationwide survey in Japan indicated marked changes in the characteristics of SCI because of the current aging of the society.^2^ This survey, which was the first of its kind to be performed in about 30 years, found that SCI was most common in patients in their 70s. Cervical SCI (CSCI) without major bone injury accounts for 70.7% of CSCI cases and is often caused by minimal trauma from events such as a fall on a level surface, which increases with age.

Paralysis after an injury is a particular concern in elderly patients because of its effects on activities of daily living (ADL). Factors affecting the prognosis for motor recovery after SCI have been identified, but few studies have focused on CSCI without major bone injury. The lack of prognostic factors for this condition leads to nonsystematic management of the condition, and there is a need for a more detailed evaluation of these factors. This study aimed to identify predictors of neurological improvement in patients aged ≥65 years with CSCI without major bone injury and to examine therapeutic interventions for increased neurological improvement.

## Methods

### Study design and ethics approval

**FIG 1.**
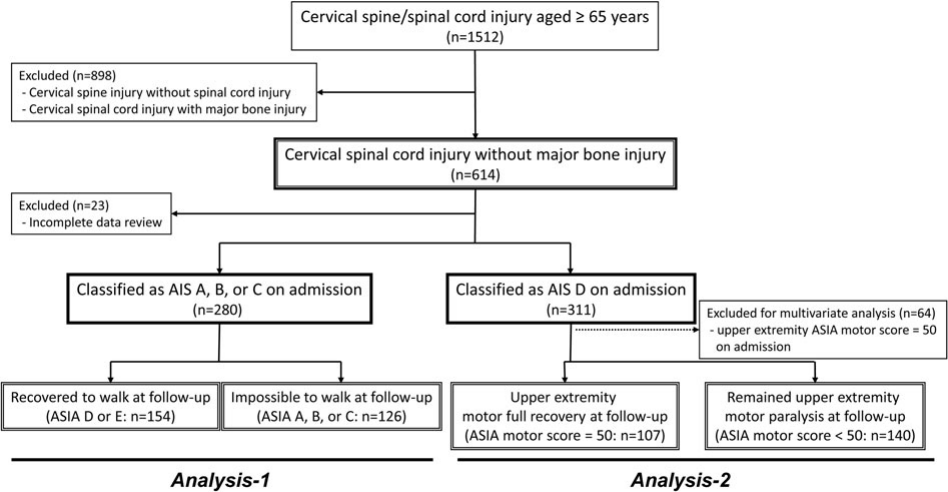
Flowchart for analyses of American Spinal Injury Association (ASIA) Impairment Scale (AIS) A, B, and C cases and AIS D cases.

A multi-center study was performed by the Japan Association of Spine Surgeons with Ambition (JASA) as a retrospective analysis of inpatients aged ≥65 years with cervical spinal cord and/or spine injury at 33 medical centers between 2010 and 2020, with a minimum follow-up period of three months. The study protocol was approved by the Institutional Review Board of the representative facility (No. 3352-1) and each center. A total of 1512 eligible patients were identified, of whom 614 (40.6%) had a diagnosis of CSCI without major bone injury; 591 of these patients were enrolled in the study (Fig. 1).

The definition of CSCI without major bone injury was SCI with no evidence of spinal fracture or dislocation on plain radiography or computed tomography.^3^ The 23 excluded patients had incomplete clinical and functional follow-up data. The following data were obtained: patient background (age, gender, body mass index [BMI]), imaging findings (presence of ossification of the posterior longitudinal ligament [OPLL]), signal change in the cervical spinal cord on T2-weighted magnetic resonance imaging (MRI) at the time of injury, cause of SCI (low-energy trauma, higher-energy trauma), diagnostic delay (> 24 h after injury), comorbidity (dementia/delirium, diabetes, hypertension), treatment (administration of methylprednisolone, operative or conservative), and post-injury complications (pneumonia, complicated injuries).

Demographic data of the patients are presented in Table 1. The mean age of participants was 75.3 years and the male:female ratio was 3:1. The rate of patients with OPLL was higher (34.3%) than the general prevalence.^4^ The most common cause of SCI was low-energy trauma caused by a fall on a level surface or from a low height.

**Table 1.** Data for 591 Patients with Cervical Spinal Cord Injury without Major Bone Injury

Item	Value
Patient background	
Age, years, mean ± SD	75.3 ± 6.7
Gender (male:female), *n* (%)	429 (72.6%):162 (27.4%)
Body mass index (kg/m^2^), mean ± SD	22.4 ± 3.6
Imaging findings	
OPLL, *n* (%)	203 (34.3%)
Signal change on MRI, *n* (%)	488 (82.6%)
Cause of spinal cord injury, *n* (%)	
Low-energy trauma	
Fall on a level surface	312 (52.8%)
Low fall (≤1 m in height)	85 (14.4%)
Higer-energy trauma	
High fall (>1 m in height)	94 (15.9%)
Traffic accident	73 (12.4%)
Others (sports-related injuries, etc.)	27 (4.6%)
Diagnostic delay, *n* (%)	68 (11.5%)
ASIA Impairment Scale (AIS) on admission, *n* (%)	
A	34 (5.8%)
B	31 (5.2%)
C	215 (36.4%)
D	311 (52.6%)
Comorbidity, *n* (%)	
Dementia/delirium	53 (9.0%)
Diabetes	165 (27.9%)
Hypertension	284 (48.1%)
Treatment, *n* (%)	
Administration of methylprednisolone	131 (22.2%)
Operation ≤24 h after injury	19 (3.2%)
>24 h after injury	279 (47.2%)
Conservative	293 (49.6%)
Post-injury complications, *n* (%)	
Pneumonia	39 (6.6%)
Complicated injuries	103 (17.4%)

SD, standard deviation; OPLL, ossification of the posterior longitudinal ligament; MRI, magnetic resonance imaging; ASIA, American Spinal Injury Association.

### Neurological evaluation

Neurological status on admission, at discharge, and six months after injury or at final follow-up (hereafter referred to as 3–6 months after injury) were evaluated by senior spine surgeons and physical therapists at each center using the American Spinal Injury Association (ASIA) impairment scale (AIS).^5^ At admission, the most frequent AIS category was D (52.6%), followed by C (36.4%), A (5.8%), and B (5.2%) (Table 1).

Mobility was also objectively assessed simultaneously (independent walking/walking with a cane/walking with a walker/standing and transferring only sitting only/unable to sit and in bed). For AIS A–C cases on admission, patients who recovered walking with or without a cane or a walker (AIS D or E) 3–6 months after injury were considered to have significant neurological improvement. For AIS D cases, considering a high prevalence of central cord syndrome (weakness in the upper extremities and less severe weakness of the lower extremities), patients without residual upper extremity motor paralysis (upper extremity AIS motor score = 50) at 3–6 months after injury were considered to have significant neurological improvement.

### Statistical analysis

Comparisons were made for AIS A–C cases and D cases with and without significant neurological improvement (Fig. 1). A subanalysis of AIS B-C cases was also performed based on the influence of data for AIS A cases on the results. Categorical variables were compared using the chi-square test, Fisher exact test, or non-parametric Mann-Whitney *U* test.

Significant factors in the univariate analysis and those reported in the literature were included in a multi-variate regression model with listwise deletion of missing data. Odds ratios (ORs) and 95% confidence intervals (CIs) were calculated to identify independent predictors of neurological improvement in CSCI without major bone injury. Statistical significance was set at *p* < 0.05. All analyses were conducted using SPSS version 24.0 (SPSS, Chicago, IL).

## Results

### Neurological status

Neurological status evaluated by AIS at admission and follow-up at 3–6 months after injury is shown in Table 2. Among AIS A–C cases at admission, 154 patients (55.0%)—11.8% in AIS A, 22.6% in AIS B, and 66.5% in AIS C—had walking recovery at follow-up. In all 591 patients, there were 13 deaths (2.2%)—8.8% in AIS A, 12.9% in AIS B, 2.3% in AIS C, and 0.3% in AIS D—within six months after injury.

**Table 2.** Changes in American Spinal Injury Association Impairment Scale between Admission and Follow-up

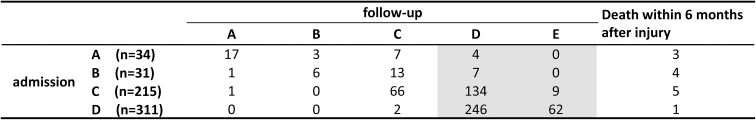

Patients in the gray area had recovery of walking at follow-up.

In AIS D cases at admission, 64 patients had a full upper extremity ASIA motor score (50). Of the remaining 247 AIS D cases, 107 (43.3%) patients recovered with full upper extremity motor function at follow-up. Among AIS C and D cases with motor paralysis, 38.6% (*n* = 83) in AIS C and 85.4% (*n* = 211) in AIS D patients were classified as having central cord syndrome.

### Prognostic factors in AIS A–C cases

A comparison of AIS A-C patients with and without recovery of walking ability at the final follow-up is shown in Table 3. In univariate analysis, the severity of AIS on admission was particularly significant, and the prevalence of OPLL, signal change on MRI, comorbidity of dementia/delirium, and post-injury pneumonia were significantly different between the patients with and without recovery of walking. There was no difference in walking recovery between the surgical and conservative treatment groups (*p* = 0.47). There were also no significant differences in patient background, cause of SCI (lower- or higher-energy trauma), diagnostic delay, comorbidity of diabetes and hypertension, administration of methylprednisolone, or complicating injuries between patients with and without walking recovery.

**Table 3.** Comparison of Admission Data for American Spinal Injury Association Impairment Scale A–C Cases with and without Independent Walking Recovery at Follow-Up

	Recovery of walking at follow-up (*n* = 154)	Could not walk at follow-up (*n* = 126)	*p*
Patient background			
Age, years, mean ± SD	75.3 ± 6.4	76.8 ± 7.2	0.15
Gender (male:female), *n*	113:41	99:27	0.31
Body mass index (kg/m^2^), mean ± SD	22.7 ± 3.6	21.9 ± 4.4	0.15
Imaging findings			
OPLL, *n* (%)	48 (31.2%)	56 (44.4%)	0.022^*^
Signal change on MRI, *n* (%)	132 (85.7%)	120 (77.9%)	0.0082^*^
Cause of spinal cord injury, *n* (%)			0.63
Low-energy trauma			
Fall on level surface	77 (50.0%)	68 (54.0%)	
Low fall (≤1 m in height)	24 (15.6%)	19 (15.1%)	
Higher-energy trauma			
High fall (>1 m in height)	28 (18.2%)	19 (15.1%)	
Traffic accident	15 (9.7%)	15 (11.9%)	
Others (sports-related injuries, etc.)	10 (6.5%)	5 (4.0%)	
Diagnostic delay, n (%)	12 (7.8%)	13 (10.3%)	0.46
ASIA Impairment Scale (AIS), n (%)			< 0.001^*^
A	4 (2.6%)	30 (23.8%)	
B	7 (4.5%)	24 (19.0%)	
C	143 (92.9%)	72 (57.1%)	
Comorbidity, n (%)			
Dementia/delirium	11 (7.1%)	24 (19.0%)	0.0027^*^
Diabetes	44 (28.6%)	45 (35.7%)	0.20
Hypertension	73 (47.4%)	58 (46.0%)	0.82
Treatment, n (%)			
Administration of methylprednisolone	37 (24.0%)	30 (23.8%)	0.97
Operation ≤24 h after injury	5 (3.2%)	13 (10.3%)	0.47
>24 h after injury	87 (56.5%)	56 (44.4%)	
Conservative	62 (40.3%)	57 (45.2%)	
Post-injury complications, *n* (%)			
Pneumonia	5 (3.2%)	30 (23.8%)	< 0.001^*^
Complicated injuries	23 (14.9%)	17 (13.5%)	0.73

SD, standard deviation; OPLL, ossification of the posterior longitudinal ligament; MRI, magnetic resonance imaging; ASIA, American Spinal Injury Association.

^*^p < 0.05.

Multi-variate logistic regression analysis including significant variables from univariate analysis and patient background factors was used to identify independent prognostic factors in AIS A-C cases (Table 4). In this analysis, BMI (OR: 1.112), signal change on MRI (OR: 0.240), AIS on admission (OR: 3.497), comorbidity of dementia/delirium (OR: 0.365), and post-injury pneumonia (OR: 0.194) were identified as independent prognostic factors for walking recovery in AIS A–C patients after CSCI without major bone injury.

**Table 4.** Multi-variate Logistic Regression Analysis of Predictors of Independent Walking Recovery in American Spinal Injury Association Impairment Scale A-C Cases after Cervical Spinal Cord Injury without Major Bone Injury

Variables	OR	95% CI	*p*
Patient background			
Age (per 1 year)	0.994	0.949–1.042	0.809
Gender (female as reference)	1.545	0.768–3.109	0.222
Body mass index (kg/m^2^)^a^ (per 1 kg/m^2^)	1.112	1.024–1.207	0.011^*^
Imaging findings			
OPLL^‡^	0.557	0.298–1.041	0.067
Signal change on MRI^‡^	0.240	0.065–0.890	0.033^*^
ASIA Impairment Scale (AIS)^†^	3.497	2.039–6.000	< 0.001^*^
Comorbidity			
Dementia/delirium ^‡^	0.365	0.140–0.953	0.040^*^
Diabetes ^‡^	0.761	0.408–1.417	0.389
Treatment			
Operation (conservative as reference)	1.043	0.758–1.436	0.794
Post-injury complications			
Pneumonia ^‡^	0.194	0.065–0.575	0.003^*^

OR, odds ratio; CI, confidence interval; OPLL, ossification of the posterior longitudinal ligament; MRI, magnetic resonance imaging; ASIA, American Spinal Injury Association.

† AIS C as reference. ^‡^ Yes vs. No.

^a^ Information missing in 20 patients.

^*^ *p* < 0.05

Multi-variate analysis excluding AIA A cases (i.e., AIS B-C cases only) showed similar results, with BMI (OR: 1.129), prevalence of OPLL (OR: 0.494), signal change on MRI (OR: 0.238), AIS on admission (OR: 4.659), and post-injury pneumonia (OR: 0.155) identified as independent prognostic factors (Table 5).

**Table 5.** Multi-variate Logistic Regression Analysis of Predictors of Independent Walking Recovery in American Spinal Injury Association Impairment Scale B and C Cases after Cervical Spinal Cord Injury without Major Bone Injury

Variables	OR	95% CI	*p*
Patient background			
Age (per 1 year)	0.999	0.951–1.049	0.971
Gender (female as reference)	1.779	0.854–3.707	0.124
Body mass index (kg/m^2^)^a^ (per 1 kg/m^2^)	1.129	1.031–1.235	0.008^*^
Imaging findings			
OPLL ^‡^	0.494	0.256–0.954	0.036^*^
Signal change on MRI ^‡^	0.238	0.063–0.899	0.034^*^
ASIA Impairment Scale (AIS) ^†^	4.659	1.947–11.147	0.001^*^
Comorbidity			
Dementia/delirium ^‡^	0.398	0.141–1.120	0.081
Diabetes ^‡^	0.797	0.416–1.526	0.493
Treatment			
Operation (conservative as reference)	0.968	0.695–1.350	0.850
Post-injury complications			
Pneumonia ^‡^	0.155	0.046–0.521	0.003^*^

OR, odds ratio; CI, confidence interval; OPLL, ossification of the posterior longitudinal ligament; MRI, magnetic resonance imaging; ASIA, American Spinal Injury Association.

† AIS C as reference. ^‡^ Yes vs. No.

^a^ Information missing in 14 patients.

^*^ *p* < 0.05

### Prognostic factors in AIS D cases

A comparison of AIS D cases with and without full upper extremity motor recovery at the final follow-up is shown in Table 6. In the univariate analysis, the severity of upper extremity ASIA motor score at admission was strongly significant. The age and comorbidity of dementia/delirium were significantly higher in patients with remaining motor paralysis at the final follow-up. In addition, significantly more patients who underwent surgery did not have full motor recovery.

**Table 6.** Comparison of Data at Admission for American Spinal Injury Association Impairment Scale D Cases with and without Full Upper Extremity Motor Recovery at Follow-Up

Variable	Motor recovery at follow-up (*n* = 107)	Motor paralysis at follow-up (*n* = 140)	*P*
Patient background			
Age, years, mean ± SD	73.7 ± 5.5	75.9 ± 7.3	0.034^*^
Gender, (male:female), *n*	71:36	103:37	0.26
Body mass index (kg/m^2^), mean ± SD	22.7 ± 3.1	22.5 ± 3.3	0.63
Imaging findings			
OPLL, n (%)	34 (31.8%)	43 (30.7%)	0.89
Signal change on MRI, *n* (%)	79 (73.8%)	117 (83.6%)	0.080
Cause of spinal cord injury, *n*			1.00
Low-energy trauma			
Fall on level surface	55 (51.4%)	74 (52.9%)	
Low fall (≤1 m in height)	16 (15.0%)	20 (14.3%)	
Higher-energy trauma			
High fall (>1 m in height)	14 (13.1%)	27 (19.3%)	
Traffic accident	18 (16.8%)	12 (8.6%)	
Others (sports-related injuries, etc.)	4 (3.7%)	7 (5.0%)	
Diagnostic delay (>24 h after injury), *n* (%)	10 (9.3%)	25 (17.9%)	0.066
Upper extremity ASIA motor score on admission	38.6 ± 7.5	32.7 ± 9.3	< 0.001^*^
Central cord syndrome, *n* (%)	94 (87.9%)	117 (83.6%)	0.37
Comorbidity, *n* (%)			
Dementia/delirium	3 (2.8%)	12 (8.6%)	0.066
Diabetes	23 (21.5%)	41 (29.3%)	0.19
Hypertension	56 (52.3%)	67 (47.9%)	0.57
Treatment, *n* (%)			
Administration of methylprednisolone	28 (26.2%)	27 (19.3%)	0.22
Operation	40 (37.4%)	73 (52.1%)	0.028^*^
Post-injury complications, *n* (%)			
Pneumonia	1 (0.93%)	3 (2.1%)	0.81
Complicated injuries	18 (16.8%)	28 (20.0%)	0.64

SD, standard deviation; OPLL, ossification of the posterior longitudinal ligament; MRI, magnetic resonance imaging; ASIA, American Spinal Injury Association.

^*^ *p* < 0.05.

There were no significant differences in gender, BMI, prevalence of OPLL, signal change on MRI, cause of SCI (lower- or higher-energy trauma), diagnostic delay, type of incomplete CSCI (central/transverse cord injury), comorbidity of diabetes and hypertension, administration of methylprednisolone, post-injury pneumonia, or complicating injuries in patients with and without motor recovery.

In multi-variate logistic regression analysis including significant variables in univariate analysis and patient background factors (Table 7), age (OR: 0.937), upper extremity ASIA motor score at admission (OR: 1.230 [per 5 scores]) and operation (OR: 0.519) were identified as independent prognostic factors for full upper extremity motor recovery in AIS D cases after CSCI without major bone injury.

**Table 7.** Multi–Variate Logistic Regression Analysis of Predictors of Full Upper Extremity Motor Recovery in American Spinal Injury Association Impairment Scale D cases after Cervical Spinal Cord Injury without Major Bone Injury

Variables	OR	95% CI	*p*
Patient background			
Age (per 1 year)	0.937	0.894–0.982	0.00658^*^
Gender (female as reference)	0.588	0.306–1.130	0.112
Imaging findings			
OPLL^†^	1.180	0.618–2.250	0.616
Signal change on MRI^†^	0.781	0.379–1.610	0.501
Diagnostic Delay (> 24 h after injury)^†^	0.583	0.238–1.430	0.238
Upper extremity ASIA motor score on admission (per 5 scores)	1.230	1.135–1.329	< 0.001^*^
Comorbidity			
Dementia/delirium^†^	0.585	0.144–2.370	0.453
Diabetes^†^	0.614	0.313–1.200	0.155
Treatment			
Administration of methylprednisolone^†^	1.490	0.727–3.040	0.277
Operation (conservative as reference)	0.519	0.272–0.990	0.0467^*^

OR, odds ratio; CI, confidence interval; OPLL, ossification of the posterior longitudinal ligament; MRI, magnetic resonance imaging; ASIA, American Spinal Injury Association.

† Yes vs. No.

^*^ *p* < 0.05

## Discussion

There has been a marked increase in the incidence of CSCI without major bone injury because of an increase in the population of elderly persons in the past 30 years in Japan. There are no reliable predictors of neurological improvement after injury, however. Therefore, we assessed 591 patients aged ≥65 years with CSCI without major bone injury using data from multiple centers.

In AIS A, B, and C cases, recovery of the ability to walk independently is the most important concern.^6–8^ Considering the high prevalence of central cord syndrome, especially in AIS D cases, recovery of upper extremity motor function is the most important concern in these patients because the usual sequence of recovery in these populations starts with motor power of the lower limbs followed by the upper limbs with fine finger movements.^9^ A neurological examination to establish the severity of injury at admission is the main prognostic factor for ambulation after SCI.^10^

In the current study, 11.8%, 22.6%, and 62.3% of AIS A, B, and C cases, respectively, recovered walking ability, and AIS severity at admission was the strongest predictor of this functional outcome. These rates were similar to those of 6.2%, 37.8%, and 69.8% in AIS A, B, and C cases, respectively, in a European multi-center study.^11^ A systematic review found rates of conversion to AIS D of 3%, 31%, and 67% for AIS A, B, and C cases, respectively, which shows that patients in the AIS A category have only a small possibility of recovery of walking.^7^

Age has previously been found to be a negative prognostic factor for walking recovery in AIS C cases: patients aged <50 years have a rate of recovery of 80–90%, but this rate is markedly reduced to 30–40% in older patients.^12,13^ In our study of patients aged ≥65 years, age had no significant effect in AIS A-C cases. In AIS D cases, however, higher age was an independent negative prognostic factor for full upper extremity motor recovery at the final follow-up. It has been suggested that rehabilitation in elderly patients should focus on functional training because these patients may find it difficult to translate neurological recovery into ADL.^14^

In the current study, BMI was also an independent predictor for recovery of walking in AIS A–C cases, consistent with the finding that an unhealthy weight (underweight and overweight) is associated with diminished functional recovery.^15^ Another multi-center study suggested that patients with obesity had a higher mortality risk within one year after SCI compared with those with normal weight, mainly because of infectious and respiratory diseases.^16^

The mechanism of CSCI without major bone injury, including central cord syndrome, is characterized by minimal trauma in patients with degenerative spinal conditions with a narrow canal from cervical spondylosis and/or OPLL. Therefore, the prevalence of a cervical narrow canal might be a risk factor for SCI, especially in Asians, who have high morbidity in such conditions.

In our study, the prevalence of OPLL of 34.3% was very high but similar to that found in a previous multi-center study in Japan.^17^ The prevalence of pre-existing factors, such as OPLL and stenosis, has also been found to be significantly higher in CSCI without bone injury than in those with bone injury in retrospective cohort studies, which suggests a link between these factors and CSCI without major bone injury.^18,19^ In our study, the prevalence of OPLL was an independent negative prognostic factor for recovery of walking ability in AIS B and C cases.

An MRI is important for the evaluation of patients with CSCI for imaging of the injured cord and prediction of outcome.^20,21^ A systematic review of MRI findings suggested that longer intramedullary hemorrhage, smaller canal diameter at maximal spinal cord compression, and the presence of spinal cord edema were associated with poor neurological recovery in acute SCI.^22^ In the current study, most patients had signal intensity changes on MRI, and AIS A–C cases with these changes had a negative impact on neurological improvement leading to walking recovery based on the results of multi-variate analysis, while there was no impact on full upper extremity motor recovery in AIS D cases.

Conventional MRI cannot distinguish recoverable from non-recoverable tissue injury, and the evidence regarding the utility of MRI in the clinical outcome of acute SCI is limited and controversial. The detailed evaluation of MRI findings (length of cord damage, T1-weighted imaging, etc.) was not assessed in the current study, but further development of MRI techniques is likely to produce more detailed prognostic findings for an injured spinal cord.

In this study, dementia and/or post-injury delirium were independent prognostic factors for the recovery of walking ability in AIS A–C cases. There is strong evidence for cognitive impairment in patients with SCI, with 40–60% showing cognitive and emotional deficits.^23^ A cross-sectional study of SCI cases indicated cognitive dysfunction in the subacute stage that worsens over time,^24^ and such cognitive and emotional impairments can compromise both quality of life and rehabilitation and recovery.

We also identified post-injury pneumonia as an independent prognostic factor in patients with AIS A–C. Associations of pneumonia, wound infection, and sepsis with poorer functional outcomes after SCI have been shown in a prospective study,^25^ and similar associations of pneumonia and/or postoperative wound infections with poorer neurological outcomes after SCI were found in AIS A–C cases in another prospective cohort study.^26^ The occurrence of post-injury pneumonia was associated with the severity of SCI, but the importance of pneumonia as a prognostic factor was identified even if AIS A cases were excluded from the current study.

Post-injury pneumonia might propagate neuronal death by promoting secondary damage and/or immune deficiency, and consequently, contribute to the restriction of neurological recovery.^27^ As in patients with dementia/delirium, this may reduce the potential for rehabilitation, which might also contribute to reduced neurological improvement. Therefore, future SCI rehabilitation should emphasize prevention of post-injury pneumonia and cognitive changes after SCI to promote functional recovery.

Most surgeons agree that surgery should be performed in patients with CSCI with bone injury within 24 h after injury,^28^ although there is controversy regarding the use of surgery or conservative management for CSCI without major bone injury. A recent randomized clinical trial on incomplete CSCI with pre-existing cervical stenosis suggested that early surgical treatment (<24 h) showed accelerated recovery within the first six months compared with delayed surgical treatment (>2 weeks).^29^

Meanwhile, a prospective study suggested that surgery was not superior to conservative treatment for patients with CSCI without major bone injury in terms of improvement of paralysis.^30^ Another prospective study found a similar absence of a significant difference in neurological improvement between the two treatment approaches and a higher frequency of post-operative complications in patients treated surgically.^31^

In the current study, the effectiveness of surgery compared with conservative treatment in requiring independent walking ability was not shown in AIS A–C cases. Further, surgical management was an independent negative factor for upper extremity motor recovery in AIS D cases. Careful consideration should be given of whether to surgically treat elderly patients with CSCI without major bone injury because the prognosis for neurological status is difficult to predict in the acute phase, and these patients are fragile and at high risk for complications. Because most of the surgically treated patients in this study had late surgery (only 19 patients received early surgery), however, no conclusion can be drawn about the impact of surgical treatment on neurological prognosis from the results of this study.

There is a need to examine whether some patients should undergo surgery for recovery of independent walking.^6^ To resolve the controversy between conservative management versus early surgery versus delayed surgery in patients with CSCI without major bone injury, there is a need for high-quality prospective randomized controlled trials.^9^

This study had several limitations, including its retrospective design, which prevented detailed evaluation of imaging findings, the inclusion of inpatients in acute-care hospitals only, and no outpatients, which may have resulted in a higher proportion of patients treated surgically, and different indications for surgical treatment including the timing and conservative treatment at each center. Within these limitations, the study in a large case series permitted evaluation of prognostic factors for neurological improvement in patients with CSCI without major bone injury, and the findings provide important insights and guidance on the treatment of such patients.

## Conclusions

The BMI, signal intensity on MRI, AIS on admission, comorbidity of dementia/delirium, and post-injury pneumonia were independently and significantly associated with walking recovery in AIS A–C cases with CSCI without major bone injury, and the prevalence of OPLL was also an independent prognostic factor in AIS B-C cases. Age, upper extremity ASIA motor score on admission, and operation were independently and significantly associated with full upper extremity motor recovery in AIS D cases. The severity of paralysis on admission has a major impact on functional outcomes, but the promotion of rehabilitation through measures to reduce cognitive changes, post-injury pneumonia, and unhealthy body weight changes can also contribute to greater neurological improvement in AIS A–C cases.

## JASA Study Group

Koji Akeda, Mie University Graduate School of Medicine, Mie, Japan; Yasuchika Aoki, Eastern Chiba Medical Center, Chiba, Japan; Haruki Funao, International University of Health and Welfare, International University of Health and Welfare Narita Hospital, Chiba, Japan; International University of Health and Welfare Mita Hospital, Tokyo, Japan; Katsumi Harimaya, Kyushu University Beppu Hospital, Oita, Japan; Yohei Haruta, Kyushu University, Fukuoka, Japan; Tomohiko Hasegawa, Hamamatsu University School of Medicine, Shizuoka, Japan; Ko Hashimoto, Tohoku University Graduate School of Medicine, Miyagi, Japan; Yoichi Iizuka, Gunma University, Graduate School of Medicine, Gunma, Japan; Shota Ikegami, Shinshu University School of Medicine, Nagano, Japan; Shiro Imagama, Nagoya University Graduate School of Medicine, Nagoya, Japan; Yasuaki Imajo, Yamaguchi University Graduate School of Medicine, Yamaguchi, Japan; Masayuki Ishihara, Kansai Medical University Hospital, Osaka, Japan; Ken Ishii, International University of Health and Welfare, International University of Health and Welfare Narita Hospital, Chiba, Japan; International University of Health and Welfare Mita Hospital, Tokyo, Japan; Kenji Kato, Nagoya City University Graduate School of Medical Sciences, Nagoya, Japan; Kenichi Kawaguchi, Kyushu University, Fukuoka, Japan; Katsuhito Kiyasu, Kochi Medical School, Kochi University, Kochi, Japan; Hideaki Matsuo, University of Fukui Hospital, Fukui, Japan; Kosuke Misaki, Kawasaki Medical School, Okayama, Japan; Hideki Murakami, Nagoya City University Graduate School of Medical Sciences, Nagoya, Japan; Kazuo Nakanishi, Kawasaki Medical School, Okayama, Japan; Hiroaki Nakashima, Nagoya University Graduate School of Medicine, Nagoya, Japan; Seiji Ohtori, Chiba University, Chiba, Japan; Seiji Okada, Osaka University Graduate School of Medicine, Osaka, Japan; Yoshito Onoda, Tohoku University Graduate School of Medicine, Miyagi, Japan; Yasushi Oshima, University of Tokyo Hospital, Tokyo, Japan; Munehiro Sakata, Kyoto Prefectural University of Medicine, Kyoto, Japan; Saiseikai Shiga Hospital, Shiga, Japan; Hirokatsu Sawada, Nihon University School of Medicine, Tokyo, Japan; Eiki Shirasawa, Kitasato University School of Medicine, Kanagawa, Japan; Nobuyuki Suzuki, Nagoya City University Graduate School of Medical Sciences, Nagoya, Japan; Hidenori Suzuki, Yamaguchi University Graduate School of Medicine, Yamaguchi, Japan; Norihiko Takegami, Mie University Graduate School of Medicine, Mie, Japan; Eiji Takasawa, Gunma University, Graduate School of Medicine, Gunma, Japan; Koji Tamai, Osaka City University Graduate School of Medicine, Osaka, Japan; Hidetomi Terai, Osaka City University Graduate School of Medicine, Osaka, Japan; Hitoshi Tonomura, Kyoto Prefectural University of Medicine, Kyoto, Japan; Masashi Uehara, Shinshu University School of Medicine, Nagano, Japan; Hiroshi Uei, Nihon University Hospital, Tokyo, Japan; Nihon University School of Medicine, Tokyo, Japan; Akihiro Yamaji, Ibaraki Seinan Medical Center Hospital, Ibaraki, Japan; Junichi Yamane, National Hospital Organization Murayama Medical Center, Tokyo, Japan; Toshitaka Yoshii, Tokyo Medical and Dental University, Tokyo, Japan; Atsushi Yunde, Chiba University, Chiba, Japan.
